# Anatomical and Functional Outcomes of Platelet-Rich Plasma-Assisted Surgery for Full-Thickness Macular Holes: A Retrospective Real-World Cohort Study

**DOI:** 10.3390/jcm15187129

**Published:** 2026-09-14

**Authors:** Hanna Haave, Beáta Éva Petrovski, Ivan Borjan, Xhevat Lumi, Goran Petrovski

**Affiliations:** 1 Center for Eye Research and Innovative Diagnostics, Department of Ophthalmology, Oslo University Hospital, 0450 Oslo, Norway; hanna_haave@hotmail.com (H.H.); b.e.petrovski@medisin.uio.no (B.É.P.); xhlumi@hotmail.com (X.L.); 2 Department of Ophthalmology, University Hospital of Split, 21000 Split, Croatia; 3 Department of Ophthalmology, Institute for Clinical Medicine, Faculty of Medicine, University of Oslo, 0450 Oslo, Norway

**Keywords:** macular hole, surgical outcomes, FTMH, platelet-rich plasma (PRP), BCVA, OCT

## Abstract

**Objectives**: To describe short-term anatomical and visual outcomes after platelet-rich plasma (PRP)-assisted surgery for full-thickness macular holes (FTMHs) in a selected real-world cohort, predominantly consisting of previously operated and large holes. **Methods**: This retrospective, register-based cohort study included 61 eyes of 61 patients who underwent FTMH surgery with adjunctive autologous PRP. Anatomical closure, short-term BCVA change, and exploratory OCT associations were evaluated overall and by surgical subgroup. **Results**: Seven eyes underwent PRP-assisted primary surgery and 54 underwent repeat surgery. Complete closure occurred in 46/61 eyes (75.4%; 95% CI, 63.3–84.5%). Median BCVA improved from 0.74 to 0.53 logMAR (*p* < 0.001); results remained directionally consistent in a 21–42-day sensitivity window (*n* = 33; *p* = 0.016). Nine eyes underwent combined phaco-vitrectomy. Preoperative MLD differed across anatomical outcomes (*p* = 0.005), where the border-cyst association with postoperative BCVA was not significant after adjustment. **Conclusions**: PRP-assisted surgery was followed by substantial short-term anatomical closure and visual improvement, but the uncontrolled design, heterogeneous management, and incomplete characterization of prior surgery and PRP preclude attribution of efficacy or safety to PRP.

## 1. Introduction

A full-thickness macular hole (FTMH) is a defect involving all retinal layers at the fovea [1]. Tangential traction along the retinal surface and anteroposterior traction at the vitreoretinal interface contribute to its development [2,3]. Because the fovea mediates high-resolution central vision, patients commonly present with metamorphopsia and reduced visual acuity. Gass et al. developed the original clinical staging system [4], and optical coherence tomography (OCT) subsequently enabled reproducible anatomical characterization through the International Vitreomacular Traction Study classification [5]. Pars plana vitrectomy, often combined with internal limiting membrane peeling and, when indicated, phacoemulsification, is the standard surgical approach and generally achieves high closure rates and meaningful visual improvement [6,7,8].

Several techniques have been developed for large, recurrent, refractory, or secondary FTMHs [9], including inverted, temporal, and free internal limiting membrane flaps [10,11,12], lens-capsule flaps [13,14], human amniotic membrane grafts [15,16], autologous retinal transplantation [17], and macular hydrodissection [18]. Autologous blood-derived adjuncts, including serum and platelet preparations, have also been investigated. Liggett et al. reported autologous serum in 1995 [19], building on experimental observations that serum-derived factors can promote cellular proliferation and migration [20,21,22,23]. Subsequent studies of blood-derived adjuncts have reported variable anatomical results [24,25,26,27], and internationally accepted indications remain undefined.

Platelets contain growth factors, including vascular endothelial growth factor, platelet-derived growth factor, and epidermal growth factor [28]. Following platelet activation, fibrin formation and growth-factor release may support cellular migration and Müller-cell responses relevant to tissue remodelling [29,30,31]. The precise biological mechanism by which a platelet concentrate might facilitate FTMH closure, however, remains uncertain.

This study describes short-term anatomical and visual outcomes after PRP-assisted FTMH surgery in a selected real-world cohort and explores whether preoperative OCT features were associated with postoperative anatomical status or BCVA. It was not designed to determine the independent efficacy or safety of PRP.

## 2. Patients and Methods

This retrospective, register-based cohort study included 61 consecutive patients with FTMH who underwent surgery with adjunctive PRP between January 2020 and June 2025 at the Department of Ophthalmology, Oslo University Hospital, Norway. FTMH was defined according to the IVTS classification as a defect involving all neural retinal layers at the fovea [5]. Clinical records were reviewed manually and entered into the quality register. Records from referring ophthalmologists were obtained when postoperative follow-up occurred outside the hospital. Eyes with a clearly secondary FTMH, including traumatic, highly myopic, or post-infectious holes, were excluded. Patient selection was based on surgeon decision to use adjunctive PRP in eyes considered clinically challenging, including persistent, recurrent, reopened, large, or otherwise complex FTMHs. The dataset included one eye per patient. During the study period, PRP was not evaluated against a contemporaneous non-PRP control group; therefore, the cohort should be interpreted as a descriptive series of PRP-assisted procedures rather than an efficacy comparison.

The study adhered to the Declaration of Helsinki and was approved as a quality-register study by the institutional data-protection authority at Oslo University Hospital in accordance with local requirements. The requirement for individual consent for retrospective review of quality-register data was waived.

**Figure 1:**
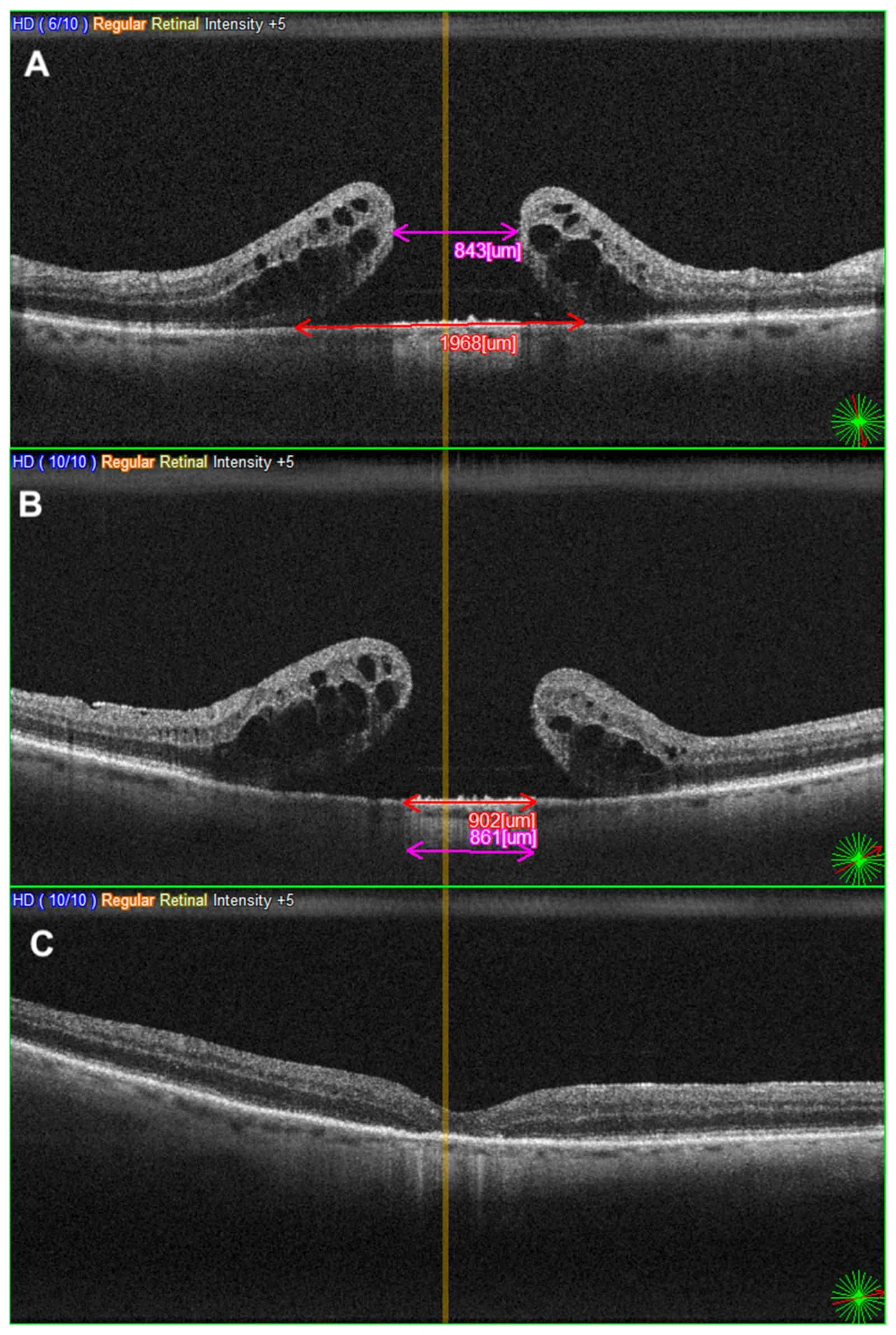
Swept-source OCT characterization of full-thickness macular holes. (**A**) Base diameter and minimum linear diameter; (**B**) retinal pigment epithelium hyperreflectivity width and choroidal hypertransmission width; and (**C**) postoperative closure in the same reoperated eye shown in panels (**A**,**B**).

Complete closure was defined as continuous neurosensory retinal tissue across the foveal defect on postoperative OCT (Figure 1). Partial closure/improvement was defined as persistent foveal discontinuity with reduction in hole configuration or flattening of the edges compared with baseline. Persistent open FTMH was defined as ongoing full-thickness foveal discontinuity without meaningful anatomical improvement. OCT grading was retrospective and unmasked; intergrader reproducibility was unavailable. An RS-3000 OCT (NIDEK Co., Ltd., version 1.7.0.4; Gamagori, Japan) was used. Manual measurements were performed on the scan judged to show the maximal foveal defect, using the full OCT volume for context when available. Scan dimensions and acquisition-density parameters were not consistently retained. Postoperative BCVA and OCT were abstracted from the same recorded short-term follow-up visit. The database did not record whether intraocular gas remained in the visual axis at BCVA testing.

Because rotor radius, relative centrifugal force, final PRP volume, and drop volume were not recorded in the retrospective database, the PRP protocol cannot be fully biologically standardized against other platelet-concentrate preparations. This limitation was considered when interpreting comparisons with prior studies.

**Figure 2:**
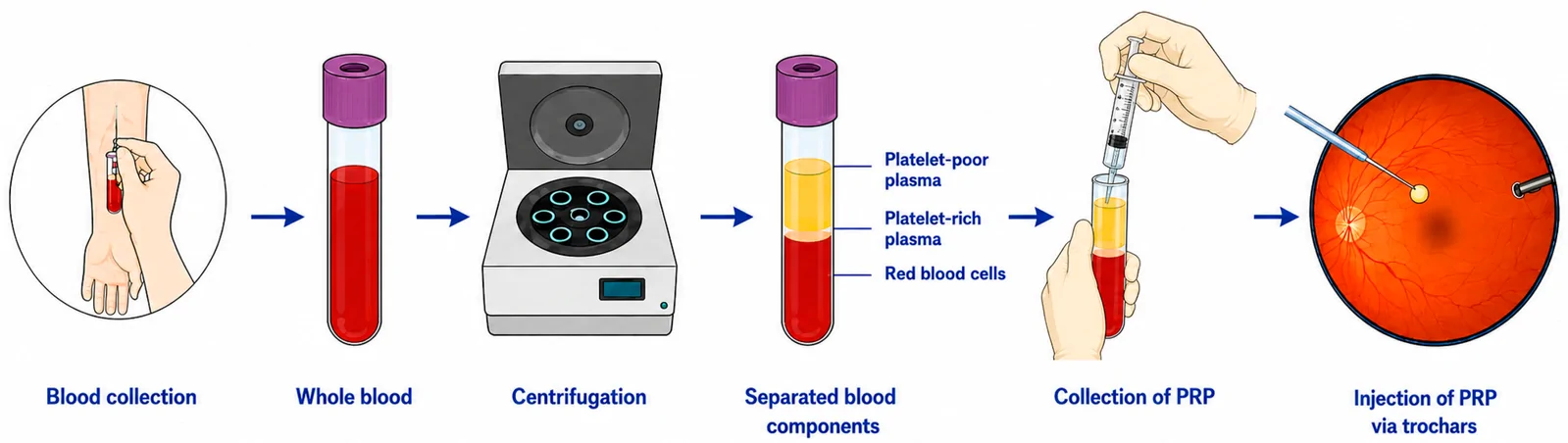
Workflow of the PRP preparation.

A single-spin liquid autologous preparation, clinically designated PRP as the platelet-rich fraction produced by the institutional protocol, was used (Figure 2). Ten millilitres of venous blood was collected into a tube containing 1 mL of 3.2% sodium citrate and centrifuged at 1600 rpm for 10 min. Three drops were placed over the macular hole after fluid–air exchange, injected intravitreally through the vitrectomy trochar. Platelet count, leukocyte concentration, red-cell contamination, enrichment factor, rotor radius, relative centrifugal force, final volume, drop volume, and growth-factor composition were not measured, and no exogenous activation was performed.

PPV was performed under retrobulbar or sub-Tenon anaesthesia. When clinically indicated, cataract surgery with intraocular lens implantation was performed before standard three-port PPV using 23- or 25-gauge instrumentation. In the primary-surgery group, core vitrectomy and posterior vitreous detachment induction were followed by ILM peeling and, when required, epiretinal membrane peeling. In the repeat-surgery group, additional ILM or epiretinal membrane peeling was considered. After fluid-air exchange, three drops of PRP were placed over the macular hole, followed by the selected endotamponade.

Patients remained supine for the first 30 min after PRP administration and then maintained a face-down position until examination on the first postoperative day. Postoperative treatment comprised topical antibiotics, corticosteroids three times daily for 3 weeks, and cyclopentolate 1% twice daily for 10 days. Follow-up with BCVA assessment and OCT was planned at 3–4 weeks, either at the hospital or with the referring ophthalmologist.

Continuous variables were summarized as mean ± standard deviation or median with interquartile range and range; categorical variables were reported as counts and percentages. Paired BCVA was compared using the Wilcoxon signed-rank test. The *n* = 7 surgery-naive analysis used an exact test and was considered exploratory because of its instability. Three-category MLD comparisons used the Kruskal–Wallis test, followed by pairwise Wilcoxon rank-sum tests with Holm adjustment. Other independent continuous and categorical comparisons used rank-based, t, chi-square, or Fisher exact tests as appropriate. All tests were two-sided.

A complete-case ordinary least-squares model evaluated postoperative BCVA as the dependent variable. Covariates were selected a priori for clinical relevance and included baseline BCVA, MLD, surgical group, phacoemulsification, follow-up duration, and intraretinal border cysts. The border-cyst coefficient represents the adjusted mean logMAR difference for cyst presence. There were no missing values for model variables. Linearity, residual distribution, homoscedasticity, leverage, and influence were assessed using residual-versus-fitted and quantile–quantile plots, leverage, and Cook distance. Except for the Holm-adjusted MLD comparisons, analyses were exploratory and unadjusted for multiplicity; nominal *p* < 0.05 was used.

Analyses were performed using SPSS version 27 (IBM), Stata version 17, and independent patient-level database verification.

## 3. Results

The cohort comprised 61 eyes of 61 patients (median age, 70 years; range, 25–87). Seven eyes (11.5%) underwent PRP-assisted primary surgery and 54 (88.5%) underwent repeat surgery (Table 1). Combined phaco-vitrectomy was performed in nine eyes (3/7 surgery-naive and 6/54 reoperated); 26 eyes were phakic preoperatively. ILM manipulation/peeling was documented in 41 eyes. Tamponades were SF6 (55.7%), C3F8 (26.2%), C2F6 (16.4%), and air (1.6%). Median follow-up was 28 days (IQR, 22–44; range, 14–274). Detailed variables describing previous macular-hole operations, prior flap techniques, prior tamponade, chronicity, and persistent versus recurrent/reopened status were not reliably available.

**Figure 3:**
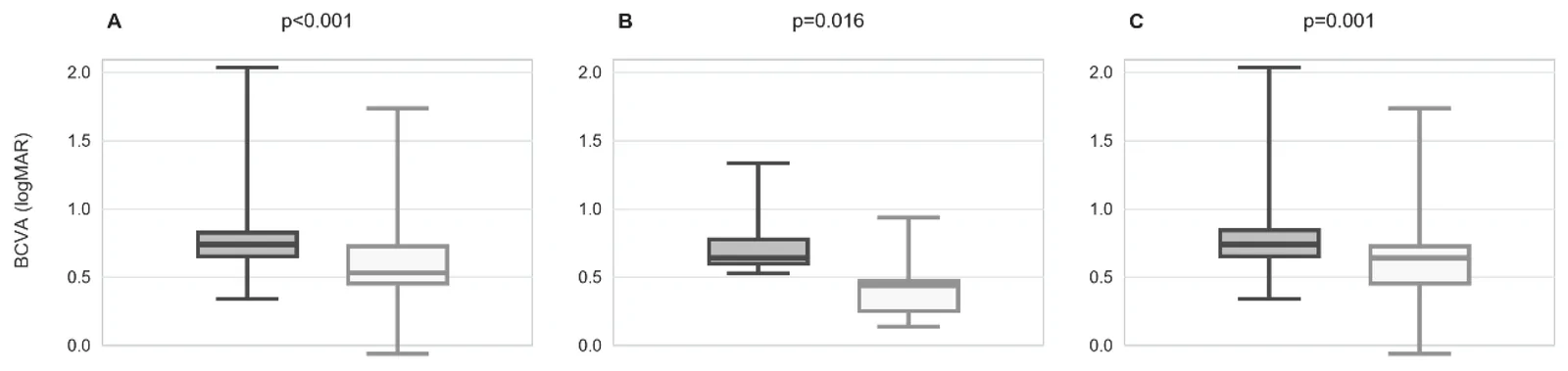
Short-term visual acuity before and after PRP-assisted surgery. Panel (**A**) shows the total cohort (*n* = 61), panel (**B**) the surgery-naive group (*n* = 7), and panel (**C**) the reoperated group (*n* = 54). Within each panel, the left dark-grey box represents preoperative BCVA and the right white box represents postoperative BCVA. Boxes show medians and interquartile ranges, and whiskers show observed ranges. *p*-values are from paired Wilcoxon signed-rank tests; the surgery-naive exact *p*-value is exploratory.

In the complete cohort, median BCVA improved from 0.74 logMAR (IQR, 0.64–0.84) to 0.53 logMAR (IQR, 0.44–0.74), with a median paired change of −0.19 logMAR (IQR, −0.32 to 0.00; *p* < 0.001) (Figure 3).

In the surgery-naive subgroup, median BCVA improved from 0.64 logMAR (IQR, 0.58–0.79) to 0.44 logMAR (IQR, 0.24–0.48; exact *p* = 0.016) (Figure 3 and Table 2). This small-subgroup inference is exploratory and unstable. In reoperated eyes, median BCVA improved from 0.74 logMAR (IQR, 0.64–0.86) to 0.64 logMAR (IQR, 0.44–0.74; *p* = 0.001) (Figure 3 and Table 2). In a sensitivity analysis limited to the predefined 21–42-day postoperative window, 33 eyes (31 reoperated) improved from 0.64 to 0.53 logMAR (*p* = 0.016). Gas status in the visual axis was not recorded, so residual-gas influence cannot be excluded.

### Anatomical Outcomes

Preoperative OCT measurements are summarized in Table 3.

The median BD was 1195 μm (IQR, 914–1391 μm), and the median MLD was 510 μm (IQR, 401–621 μm). Median central macular thickness was 326 μm (IQR, 263–415 μm), median retinal pigment epithelium hyperreflectivity width was 571 μm (IQR, 486–692 μm), and median choroidal hypertransmission width was 533 μm (IQR, 427–653 μm).

**Figure 4:**
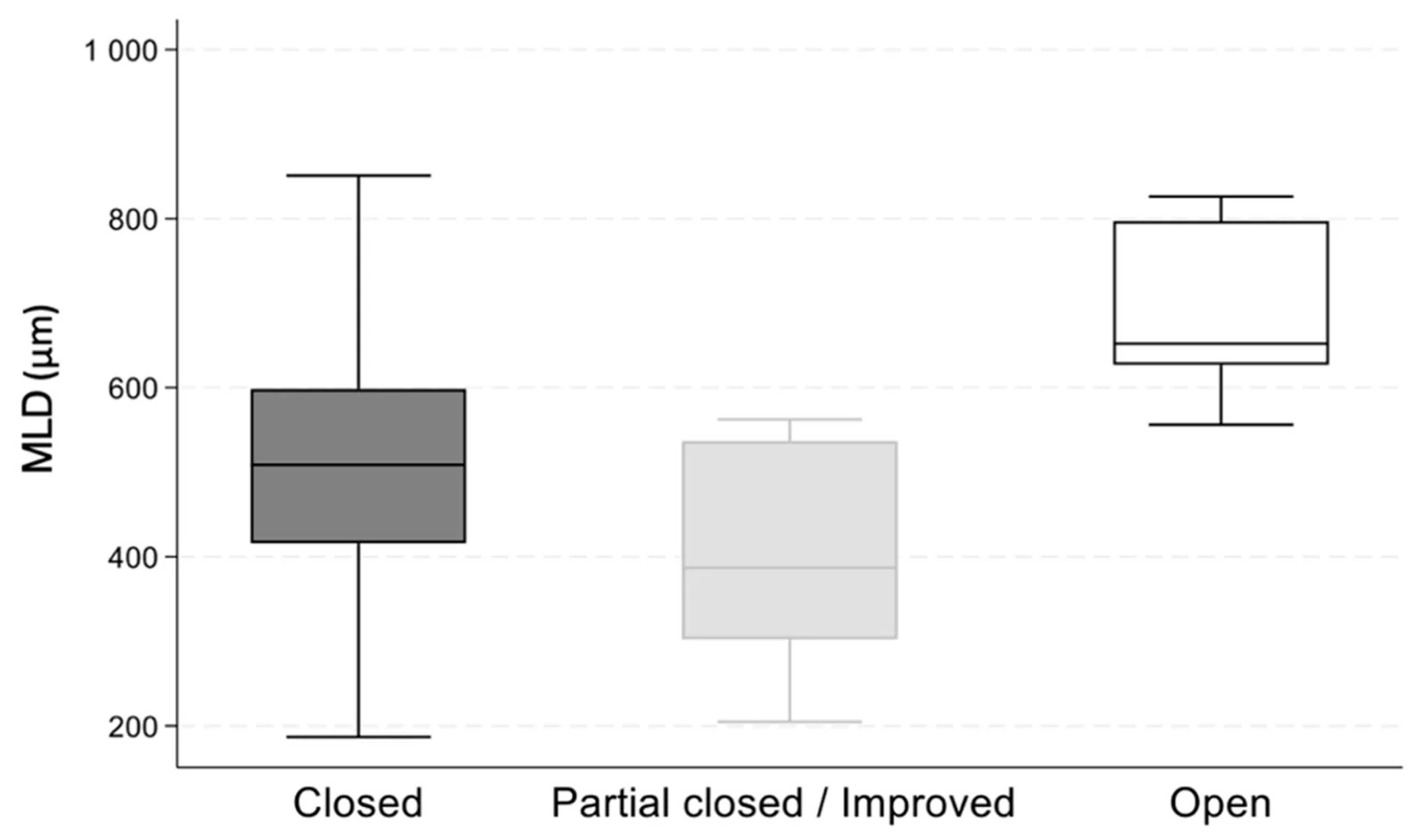
Minimum linear diameter according to postoperative anatomical outcome. Box plots compare preoperative MLD among eyes with complete closure, partial closure or anatomical improvement, and persistent open full-thickness macular holes.

Complete anatomical closure was achieved in 46 of 61 eyes (75.4%; 95% CI, 63.3–84.5%). Partial closure or anatomical improvement was observed in eight eyes (13.1%), and seven eyes (11.5%) remained open. Complete closure occurred in 6/7 surgery-naive eyes (85.7%; 95% CI, 48.7–97.4%) and 40/54 reoperated eyes (74.1%; 95% CI, 61.1–83.9%). The distribution of MLD according to postoperative anatomical status is shown in Figure 4.

Median MLD differed significantly among eyes with complete closure (508.5 μm), partial closure or anatomical improvement (387 μm), and persistent open macular holes (652 μm; Kruskal–Wallis *p* = 0.005). Post-hoc pairwise comparisons with Holm correction indicated higher MLD in persistent open holes than in completely closed holes (adjusted *p* = 0.009) and partially closed/improved holes (adjusted *p* = 0.048), whereas the difference between completely closed and partially closed/improved holes was not significant (adjusted *p* = 0.169). This exploratory, unadjusted pattern does not establish MLD as an independent predictor of PRP-assisted closure.

**Figure 5:**
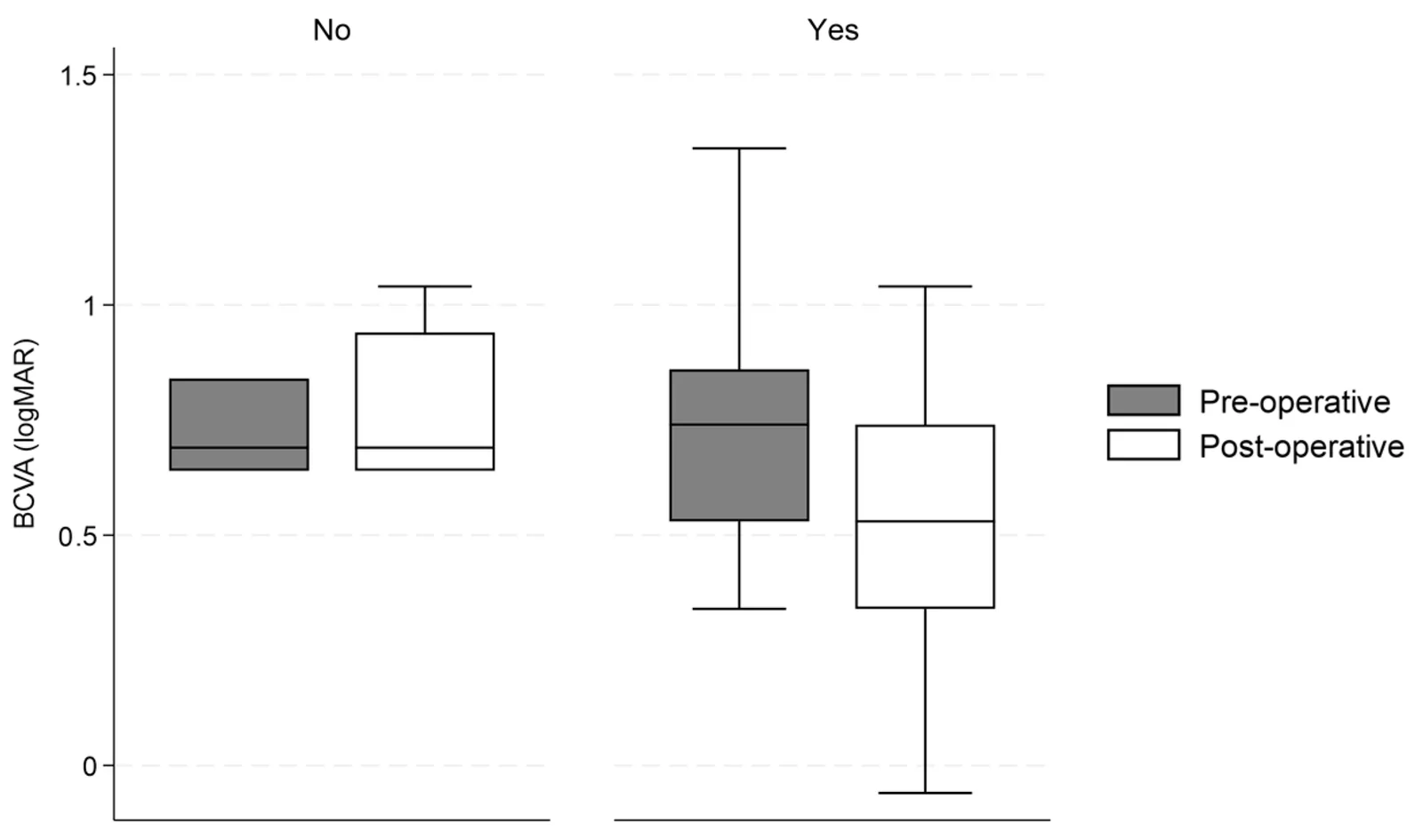
Intraretinal border cysts and visual acuity. Preoperative and postoperative BCVA distributions are shown for eyes without and with intraretinal border cysts; the unadjusted postoperative association was attenuated after multivariable adjustment.

The relationship between preoperative intraretinal border cysts and BCVA is shown in Figure 5.

Intraretinal border cysts were not associated with postoperative anatomical status (*p* = 1.00) or preoperative BCVA (*p* = 0.67). In the unadjusted analysis, postoperative BCVA was better in eyes with cysts (median, 0.53 vs. 0.69 logMAR; *p* = 0.038). After adjustment for baseline BCVA, MLD, surgical group, phacoemulsification, and follow-up, the association was attenuated (adjusted beta, −0.17 logMAR; 95% CI, −0.42 to 0.08; *p* = 0.17). This exploratory result does not establish an independent biomarker.

**Figure 6:**
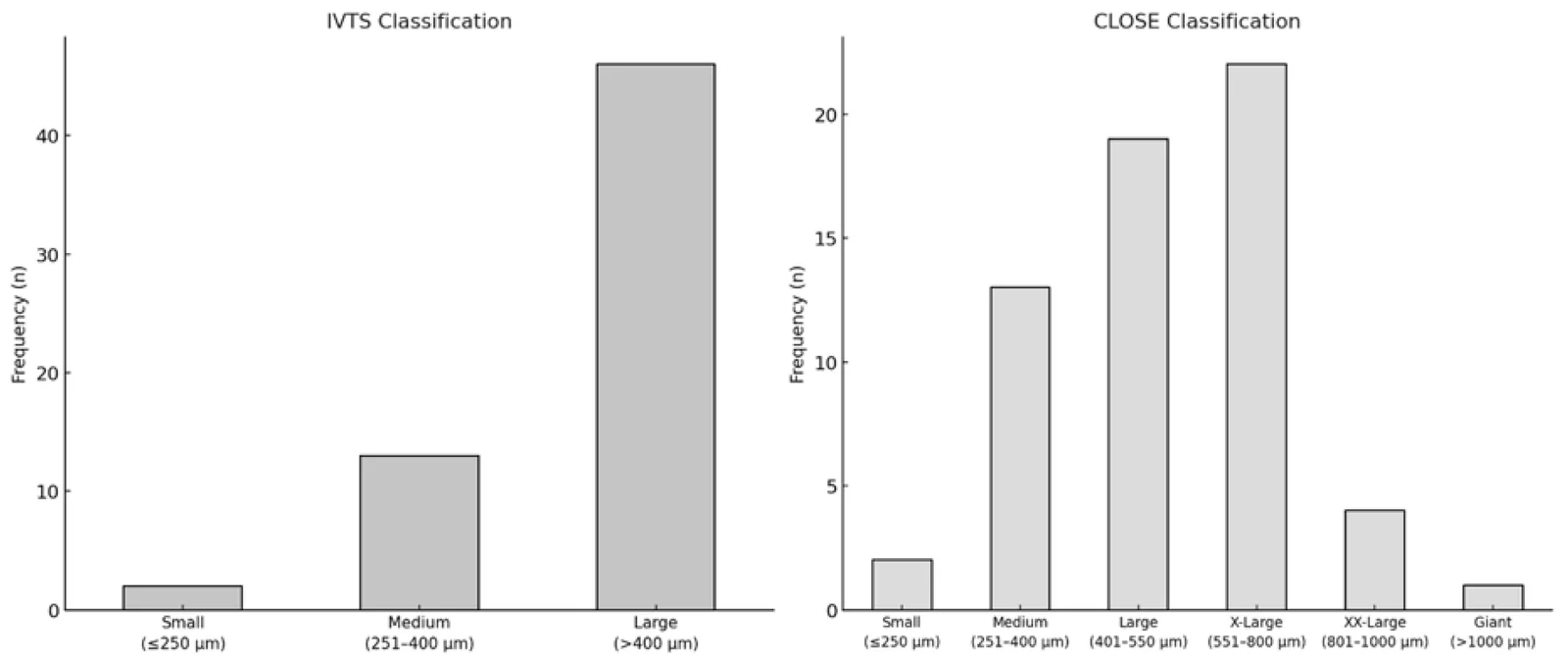
Macular-hole size distribution according to OCT classifications. The left panel shows IVTS categories and the right panel shows the more granular CLOSE categories based on minimum linear diameter.

The CLOSE classification subdivided the cohort into two small holes (3.3%), 13 medium holes (21.3%), 19 large holes measuring 401–550 µm (31.1%), 22 X-large holes measuring 551–800 µm (36.1%), four XX-large holes measuring 801–1000 µm (6.6%), and one giant hole measuring >1000 µm (1.6%). Overall, 27 eyes (44.3%) had an MLD exceeding 550 µm, demonstrating that the cohort contained a substantial proportion of anatomically complex macular holes (Figure 6). IVTS classification is shown accordingly.

**Figure 7:**
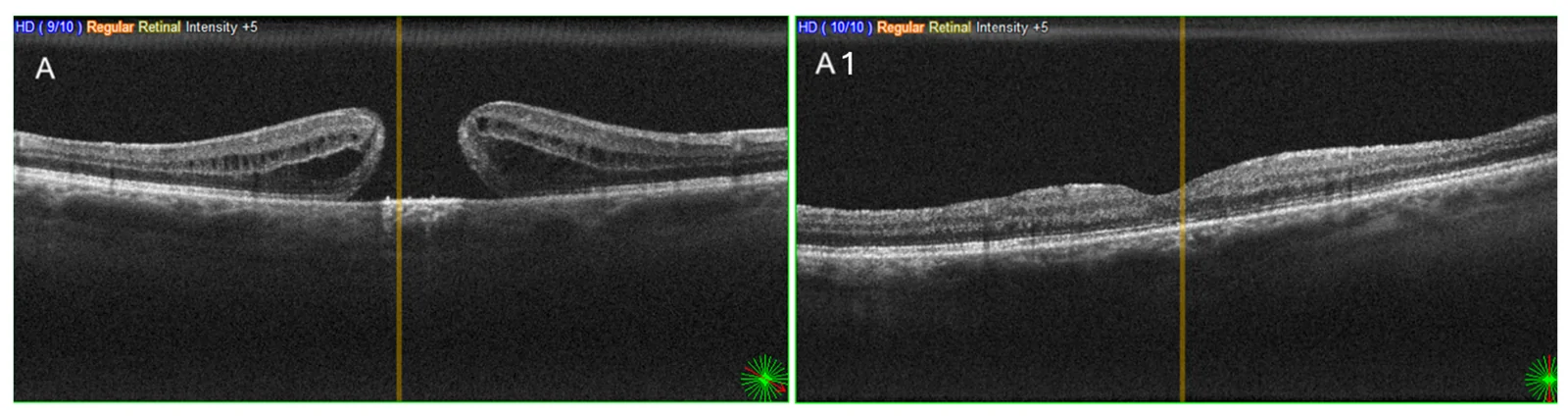

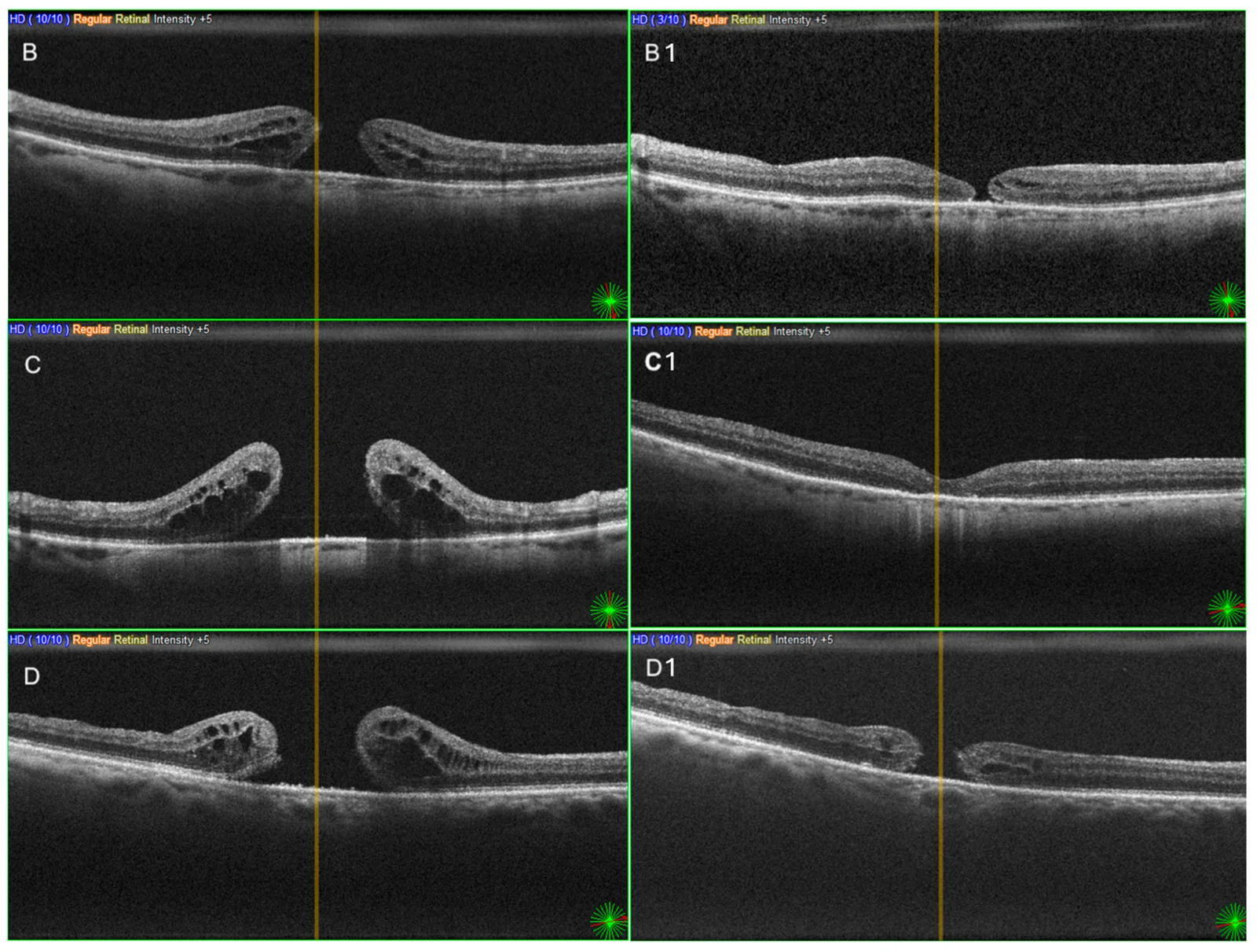
Representative preoperative and postoperative OCT outcomes after PRP-assisted surgery. Preoperative images are shown on the left (**A**) and postoperative images on the right (**A1**): surgery-naive eye with complete closure; (**B**) surgery-naive eye with (**B1**) partial closure/improvement; (**C**) reoperated eye with (**C1**) complete closure; and (**D**) reoperated eye with (**D1**) persistent open FTMH.

Representative preoperative and postoperative OCT images are presented in Figure 7. They illustrate complete closure in a surgery-naive eye, partial closure/improvement in a surgery-naive eye, and complete closure and persistent opening in reoperated eyes. No endophthalmitis, retinal detachment, clinically documented excessive inflammation, vitreous or retinal haemorrhage, proliferative vitreoretinopathy, or PRP-specific adverse events were identified in the retrospective records. These observations are descriptive and do not establish safety.

## 4. Discussion

Autologous platelet preparations have been studied in FTMH surgery since the mid-1990s [19,32]. A multicentre double-masked randomized trial reported higher anatomical closure with autologous platelet concentrate than with surgery alone, although visual outcomes did not differ significantly [33]. Subsequent studies have yielded variable results [24,25,26,27,34,35]. Because these studies differ in case mix, platelet preparation, internal limiting membrane strategy, tamponade, and follow-up, the present cohort should not be interpreted as evidence of superiority or incremental PRP efficacy.

Preoperative MLD differed across anatomical outcomes (Kruskal–Wallis *p* = 0.005). Holm-adjusted pairwise comparisons showed higher MLD in persistent open holes than in completely closed holes (*p* = 0.009) and partially closed/improved holes (*p* = 0.048); complete and partial outcomes did not differ (*p* = 0.169). Because the partial group was small and no adjusted closure model was feasible, MLD should not be considered an independent predictor.

The CLOSE Study Group proposed granular size categories because the IVTS > 400-μm category contains holes with different surgical prognoses [36]. Outcome reporting based on the IVTS classification has likewise shown clinically relevant variation within size strata [37]. In this cohort, 27 eyes (44.3%) exceeded 550 μm. These classifications describe case mix but do not justify direct efficacy comparisons across heterogeneous prior surgery, techniques, and follow-up.

Ruban et al. recently proposed foveal actual defect (FAD) as an OCT biomarker that incorporates the base diameter and lengths of detached nasal and temporal retina [38]. Their categories were small (≤200 µm), medium (201–400 µm), and large (>400 µm). FAD may offer additional prognostic information beyond MLD, but external validation and direct comparison across surgical strategies are needed before routine adoption.

PRP has also been studied in persistent and refractory FTMHs. Figueroa et al. reported closure in two highly myopic refractory cases [39], and a later series evaluated larger cohorts [25,35,40,41,42]. Degenhardt et al. reported closure in 62 of 103 eyes (60.2%) after repeat vitrectomy with autologous platelet concentrate [42]. In a multicentre prospective study of large, recurrent, or highly myopic holes, Kim et al. observed closure in 89.7% of PRP-treated eyes and 79.7% of controls at 6 months; the between-group difference was not statistically significant (*p* = 0.134) [35]. These heterogeneous findings reinforce the need for adequately powered, standardized comparative trials.

Eyes with intraretinal border cysts had better postoperative BCVA in an unadjusted analysis. Figueroa et al. similarly reported cystoid spaces as a positive prognostic factor in highly myopic holes [25]. Nevertheless, the present association may reflect baseline anatomy, chronicity, or other confounding factors and should not be interpreted as an independent predictive biomarker without multivariable validation.

Ellipsoid-zone discontinuity was more frequent among eyes with border cysts, although the association did not reach statistical significance. Neither ellipsoid-zone discontinuity nor RPE hyperreflectivity was associated with postoperative BCVA in this cohort. These null findings are imprecise because of the small sample and should not be taken as evidence that the biomarkers lack prognostic value [43].

Previous work has associated greater choroidal hypertransmission width with worse postoperative BCVA and reported that it explained more variance in final visual outcome than base diameter or MLD [44]. No significant association was detected in this cohort. Differences in sample composition, measurement, follow-up, and statistical power may account for the discrepancy.

Platelet preparations differ in centrifugation protocol, platelet dose, leukocyte content, fibrin architecture, anticoagulants, and activation [45]. This heterogeneity limits reproducibility and cross-study comparisons. Studies comparing platelet concentrate with whole blood have generally reported higher closure rates with platelet concentrate [46,47], but these non-equivalent preparations should not be treated as interchangeable. The present study used liquid autologous PRP without an added activator; platelet concentration and leukocyte content were not measured. A recent systematic review and meta-analysis of comparative studies reported higher odds of anatomical closure with autologous platelet concentrate [48], but the included studies used heterogeneous platelet preparations and surgical protocols. This reinforces the need for standardized platelet-product reporting and controlled prospective designs.

Limitations include the retrospective single-centre design; only seven surgery-naive eyes; no concurrent comparator; unavailable detailed prior-operation history; heterogeneous techniques, surgeons, and tamponades; short and heterogeneous follow-up; unknown residual-gas status during BCVA measurement; incompletely retained OCT acquisition parameters; retrospective unmasked OCT grading without reproducibility assessment; incomplete PRP characterization; retrospective safety ascertainment; and limited multivariable power. The study cannot establish PRP efficacy or safety.

## 5. Conclusions

In this selected uncontrolled cohort, PRP-assisted FTMH surgery was followed by complete closure in 75.4% of eyes and short-term BCVA improvement. The 21–42-day sensitivity analysis was directionally consistent, but heterogeneous surgery, prior treatment, follow-up, and unknown residual-gas status prevent attribution to PRP. MLD and border-cyst findings remain exploratory and require validation. Prospective controlled studies should standardize surgery and platelet products, document prior procedures and OCT acquisition, prespecify grading and analyses, and assess longer-term safety.

## Figures and Tables

**Table 1:** Baseline, surgical, and short-term postoperative characteristics of the study population.

	Naive (*n* = 7)	Reoperated (*n* = 54)
	**Age (years; median)**	
	70.0	69.5
	**Gender**	
**Male**	2	22
**Female**	5	32
	**Surgical procedures**	
**Phacoemulsification**	3	49 6
**ILM-peeling**	7	34
**Baseline BCVA, logMAR, median (IQR)**	0.64 (0.58–0.79)	0.74 (0.64–0.85) 0.74 (0.64–0.86)
**Postoperative BCVA, logMAR, median (IQR)**	0.44 (0.24–0.48)	0.64 (0.44–0.74)
**MLD, μm, median (IQR)**	568 (402–630)	510 (412–621)
**BD, μm, median (IQR)**	1072 (994–1228)	1207 (910–1410)
**Preoperative phakic lens status**	6	20
**ERM present**	4	25
**Postoperative follow-up, days, median (IQR)**	22 (20–41)	28 (22–45)
**Complete closure**	6	40
**Partial closure/improvement**	1	7
**Persistent open FTMH**	0	7

Abbreviations: *n*, number; ILM, internal limiting membrane.

**Table 2:** Summary of preoperative and postoperative BCVA (logMAR).

	Timepoint	Median (IQR)	Range (Min–Max)	Mean ± SD	*p* -Value ^†^
**Total**	Preop	0.74 (0.64–0.84)	0.34–2.04	0.78 ± 0.30	
	Postop	0.53 (0.44–0.74)	−0.06–1.74	0.59 ± 0.30	<0.001
**Surgery-naive**	Preop	0.64 (0.58–0.79)	0.53–1.34	0.75 ± 0.28	
	Postop	0.44 (0.24–0.48)	0.14–0.94	0.42 ± 0.27	0.016 *
**Reoperated**	Preop	0.74 (0.64–0.86)	0.34–2.04	0.79 ± 0.31	
	Postop	0.64 (0.44–0.74)	−0.06–1.74	0.61 ± 0.30	0.001

^†^ Wilcoxon signed-rank test comparing paired preoperative and postoperative BCVA. * The surgery-naive subgroup (*n* = 7) is reported descriptively because reliable subgroup inference is limited at this sample size. Abbreviations: BCVA, best-corrected visual acuity; IQR, interquartile range; logMAR, logarithm of the minimum angle of resolution; and SD, standard deviation. *p*-values are from paired Wilcoxon signed-rank tests; the surgery-naive exact result is exploratory.

**Table 3:** Preoperative OCT anatomical measurements.

	Median (µm)	IQR (µm)	Range (µm)	Mean ± SD (µm)
**Base Diameter (BD)**	1195	914–1391	574–3042	1244.1 ± 456.9
**Minimum Linear Diameter (MLD)**	510	401–621	187–1031	530.2 ± 169.0
**Central Macular Thickness (CMT)**	326	263–415	39–689	338.1 ± 117.2
**RPE Hyperreflectivity Width**	571	486–692	234–1078	591.9 ± 165.7
**Choroidal Hypertransmission Width (CHW)**	533	427–653	210–996	534.7 ± 166.4

## Data Availability

The original contributions presented in this study are included in the article. Further inquiries can be directed to the corresponding author.
